# MiR-429 reverses epithelial-mesenchymal transition by restoring E-cadherin expression in bladder cancer

**DOI:** 10.18632/oncotarget.8557

**Published:** 2016-04-02

**Authors:** Chia-Lun Wu, Jar-Yi Ho, Sheng-Chieh Chou, Dah-Shyong Yu

**Affiliations:** ^1^ Graduate Institute of Life Science, National Defense Medical Center, Taipei, Taiwan; ^2^ Department of Pathology, and Graduate Institute of Pathology and Parasitology, Tri-Service General Hospital, National Defense Medical Center, Taipei, Taiwan; ^3^ Division of Urology, Department of Surgery, Armed Forces Taoyuan General Hospital, Taoyuan, Taiwan; ^4^ Uro-Oncology Laboratory, Division of Urology, Department of Surgery, Tri-Service General Hospital, National Defense Medical Center, Taipei, Taiwan

**Keywords:** bladder cancer, microRNA-429, epithelial-mesenchymal transition, E-cadherin, urothelial cell carcinoma

## Abstract

Epithelial-mesenchymal transition (EMT) accompanying loss of E-cadherin is important for invasiveness and metastasis of bladder cancer. MicroRNAs (miRs) had been associated with cancer progression and differentiation in several cancers. Our goal is to find out the specific miR which modulates EMT in bladder cancer. Real-time quantitative polymerase chain reaction was used to measure the miRs expression in urothelial cell carcinoma (UCC) cell lines. MiR or siRNA mimics was used to regulate miR and mRNA level respectively. Migration and scratch assays were used to determine the migratory ability. Zymography assay was used to confirm the metalloproteinase activity. Western blotting was used to elucidate the mechanism which regulated by specific miR. MiR-429 was highly expressed in low grade UCC cell lines. Exogenous mimic of miR-429 treatment dramatically inhibited the migratory ability of T24 cells. MiR-429 downstream target ZEB1 was decreased, E-cadherin was restored, and β-catenin was contrarily decreased by exogenous mimic of miR-429 treatment in T24 cells. Cell invasive ability was also inhibited by exogenous mimic of miR-429 treatment through inactivating the MMP-2 activity in T24 cells. E-cadherin protein expression level was inhibited by E-cadherin siRNA accompanied with increasing cell migratory ability when compared with control group in low grade TSGH8301 cells. MiR-429 decreased the cell migratory and invasive abilities through reducing ZEB1 and β-catenin, restoring the E-cadherin expression and inactivation of MMP-2 of UCC cells. MiR-429 might be used as a progression marker of bladder cancer.

## INTRODUCTION

Cancer ranks as the leading causes of mortality in Taiwan. Totally 2,003 people were first diagnosed with bladder cancer and accompanied 807 (40.3% mortality rate) deaths in 2012 [1]. Approximately 30% of patients with papillary tumors of bladder will progress to invasive UCC, whereas radical cystectomy is the standard therapy. Unfortunately, this disease recurs in up to 50% of these patients despite surgery, and is potentially lethal. Half of the patients with muscle invasive bladder carcinoma will develop into metastatic disease accompanied about 90% mortality rates [2].

Epithelial-mesenchymal transition (EMT) is a key process that plays essential roles in cancer cell invasion and migration. EMT is characterized by loss of epithelial cadherin (E-cadherin) and increased expression of several transcriptional repressors of E-cadherin expression, such as zinc finger E-box-binding homeobox 1 (ZEB1) and zinc finger E-box-binding homeobox 2 (ZEB2) [3]. Matrix metalloproteinase (MMP)-2 is the main enzymes involved in extra cellular matrix (ECM) degradation during EMT process, and considered to be important in tumor invasion and distal metastasis through blood vessel or lymphoid system in bladder cancer [4].

MicroRNAs (miRs), a class of small, non-coding RNAs with 18–24 mers of nucleotides, have been shown to play a role in regulating gene expression. Mature miRs down regulate gene expression by binding the 3′-UTR region of the target gene and cause translational inhibition or mRNA degradation ultimately moderate protein expression level [5]. MiR-200 family has been reported as tumor suppressor miRNAs and significantly involved in inhibition of EMT, reduction of cancer stem cells self-renewal, modulation of cell proliferation and apoptosis, and reversal of chemoresistance in several cancer types [6]. The miR-200 family includes five members, miR-200a, miR-200b, and miR-429 are located on chromosome 1 and miR-200c and miR-141 are on chromosome 12, and several EMT genes have been reported as direct targets of miR-200 family members. In bladder cancer miR-200b and miR-200c have been reported to reduce expression of ZEB1, ZEB2, and ErbB receptor inhibitor-1 and therefore inhibit EMT and restore epidermal growth factor receptor dependency [7]. MiR-200c also target polycomb complex protein BMI-1 and E2F transcription factor 3 (E2F3) to inhibit bladder cancer cell migration and proliferation [8]. MiR-200c-141 and ZEB1/2 are also known to reciprocal repression of transcription in a negative feedback loop. MiR-200c and miR-205 coordinate epigenetic repression by Twist1 in invasive bladder cancer [9]. Accordingly, the biological functions of miR-429 have not been addressed in bladder cancer.

This study is designed to realize the role of miR- 429 in cancer progression of bladder cancer. Furthermore, we would analyze the biological and molecular changes which regulated by miR-429 to understand its underlying mechanism in bladder cancer.

## RESULTS

### Different miR-429 and E-cadherin expression in UCC cell lines

**Figure 1:**
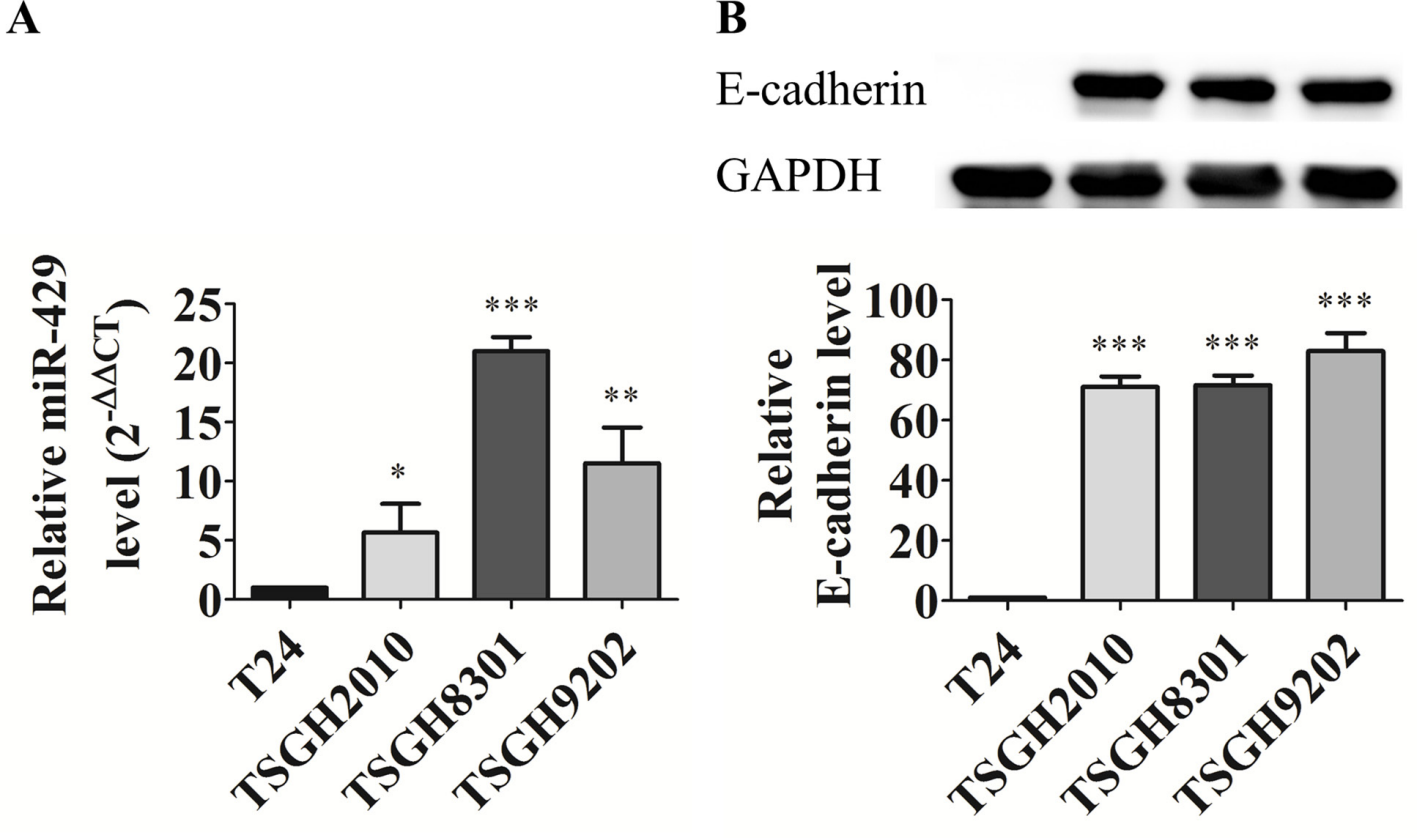
Different miR-429 and E-cadherin expression in UCC cell lines (**A**) Relative miR-429 level. Using U6 as a loading control, data was quantified with 2^−ΔΔCT^ method and T24 cell was used as reference group. (**B**) Western blotting and relative E-cadherin level (E-cadherin/GAPDH, 100X) in high grade T24 and TSGH2010 and low grade TSGH8301 and TSGH9202 UCC cell lines, statistical analysis was showed as histogram graph (Student *t*-test, *p < 0.05; **p < 0.01; ***p < 0.001, Error bars = SD).

After screening of 42 EMT-related miRs [summarized from reviews in [6, 10, 11], we found the expression of members of miR-200 family, such as miR-200b, miR-200c and miR-429, and several miR-200-associated miRNAs, such as miR-203, miR-205, and miR-206, are significantly distinguishable between distinct differential UCC cell lines (data not shown). The endogenous expression of miR- 429 was also compared among two low grade and two high grade UCC cell lines. MiR-429 is higher expressed in low grade UCC cell lines, TSGH8301 (21.0 ± 1.2 fold; *p* < 0.001) and TSGH9202 (11.5 ± 3.0 fold; *p* < 0.01) than high grade UCC cell line, TSGH2010 (5.7 ± 2.4 fold; *p* < 0.05) and T24 (possessed lowest miR-429 expression pattern and designated as comparative baseline) (Figure 1A). E-cadherin is higher expressed in high-miR-429-expressed UCC cells whereas there is no E-cadherin detectable in low-miR-429-expressed T24 cells (Figure 1B).

### Exogenous miR-429 regulates migratory ability of T24 cells

**Figure 2:**
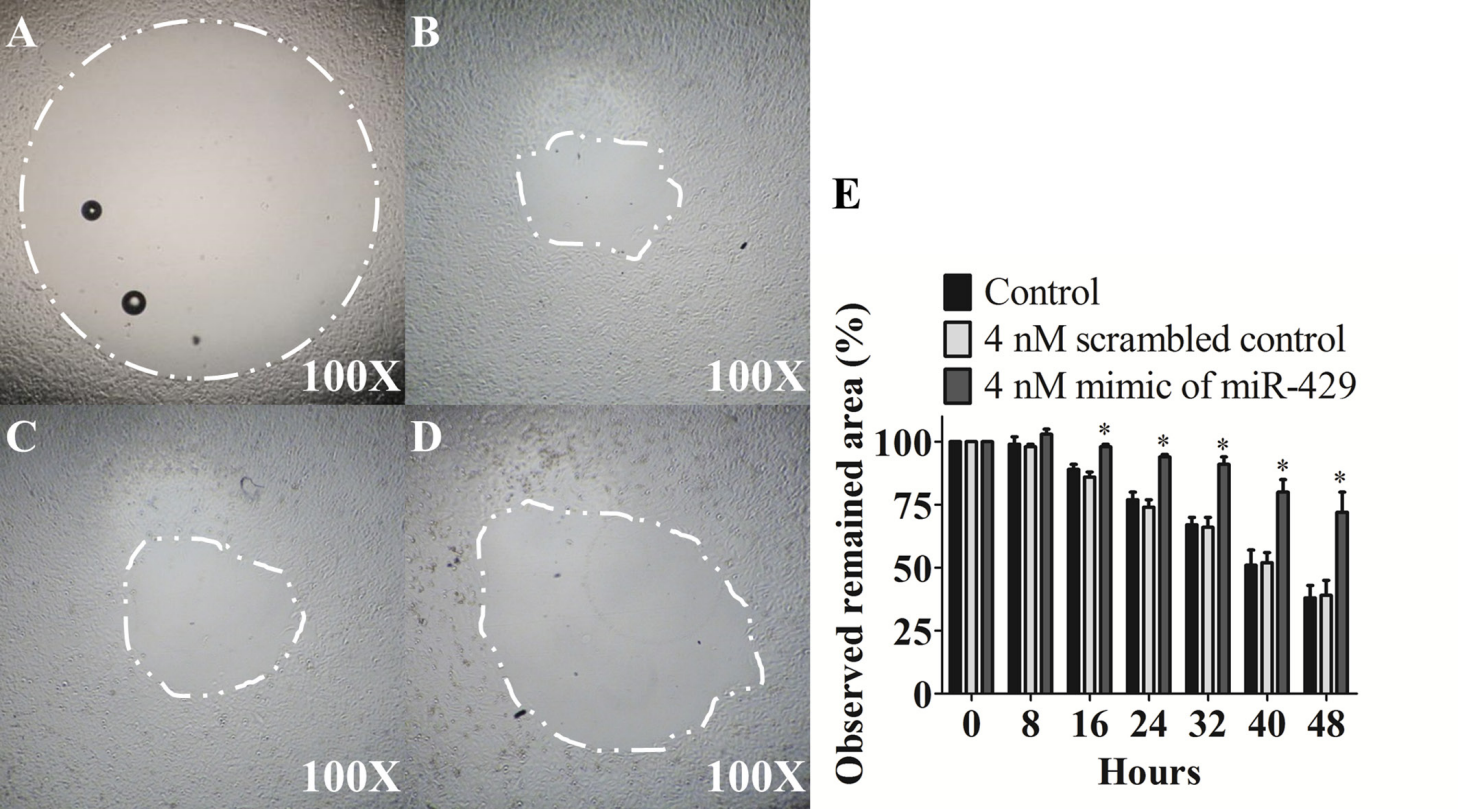
Exogenous miR-429 regulates migratory ability of T24 cells Migration assay of observed remained area of (**A**) control 0 hour, (**B**) control 48 hours, (**C**) 4 nM scrambled control 48 hours, (**D**) 4 nM mimic of miR-429 48 hours in T24 cells and (**E**) statistical analysis was showed as histogram graph (Student *t*-test, *p < 0.05, Error bars = SD).

T24 cells were transfected with 4 nM mimic of miR-429 and 4 nM scrambled control to compare their migratory abilities of 48 hours. The observed remained area shows that 4 nM of exogenous miR-429 dramatically inhibited cell migratory ability than scrambled control (71.6% ± 4.4% vs 39.1% ± 3.4%, *p* < 0.05) and non-treated control groups (71.6% ± 4.4% vs 38.3% ± 2.4%, *p* < 0.05) in T24 cells (Figure 2). These results suggest that miR-429 has potential to inhibit cell migratory ability of UCC cells.

### MiR-429 modifies E-cadherin expression in UCC cell line T24 through ZEB1-β-catenin axis in T24 cells

**Figure 3:**
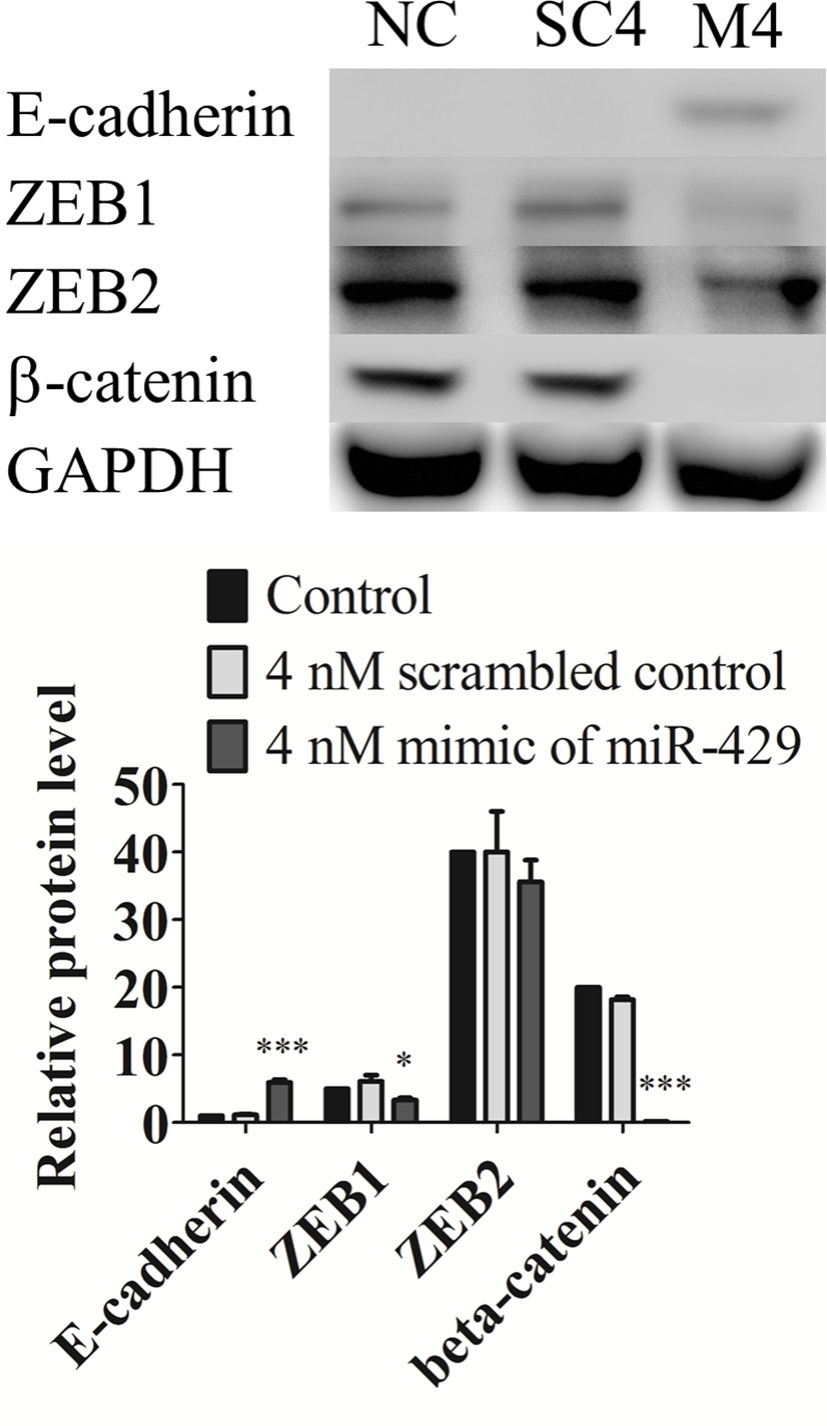
MiR-429 modifies E-cadherin expression in UCC cell line T24 through ZEB1-β-catenin axis in T24 cells Western blotting of E-cadherin, ZEB1, ZEB2, and beta-catenin was performed in three groups, negative control (NC), 4 nM scrambled miRNA control (SC), and 4 nM mimic of miR-429 (M) in T24 cells. GAPDH was used as loading control. Statistical analysis was showed as histogram graph histogram graph (Student *t*-test, *p < 0.05; ***p < 0.001, Error bars = SD).

To evaluate miR-429 role in EMT regulation, T24 cells were transfected with 4 nM mimic of miR- 429 and 4 nM scrambled control to assay EMT related proteins expression. MiR-429 targets, ZEB1 (0.67 ± 0.06, *p* < 0.05) and ZEB2 (0.89 ± 0.08, *p* = 0.178) were decreased in 4 nM exogenous miR-429 group when compared with scrambled control group. E-cadherin, downstream protein of ZEB1 and ZEB2, was dramatically restored (5.93 ± 0.42, *p* < 0.001) and β-catenin (0.01 ± 0, *p* < 0.001) was contrarily decreased when compared with scrambled control group (Figure 3). These results suggest that miR-429 inhibits ZEB1 and subsequence restores E-cadherin expression and down regulates β-catenin in UCC cells.

### Invasive ability is inhibited by exogenous miR- 429 in T24 cells

**Figure 4:**
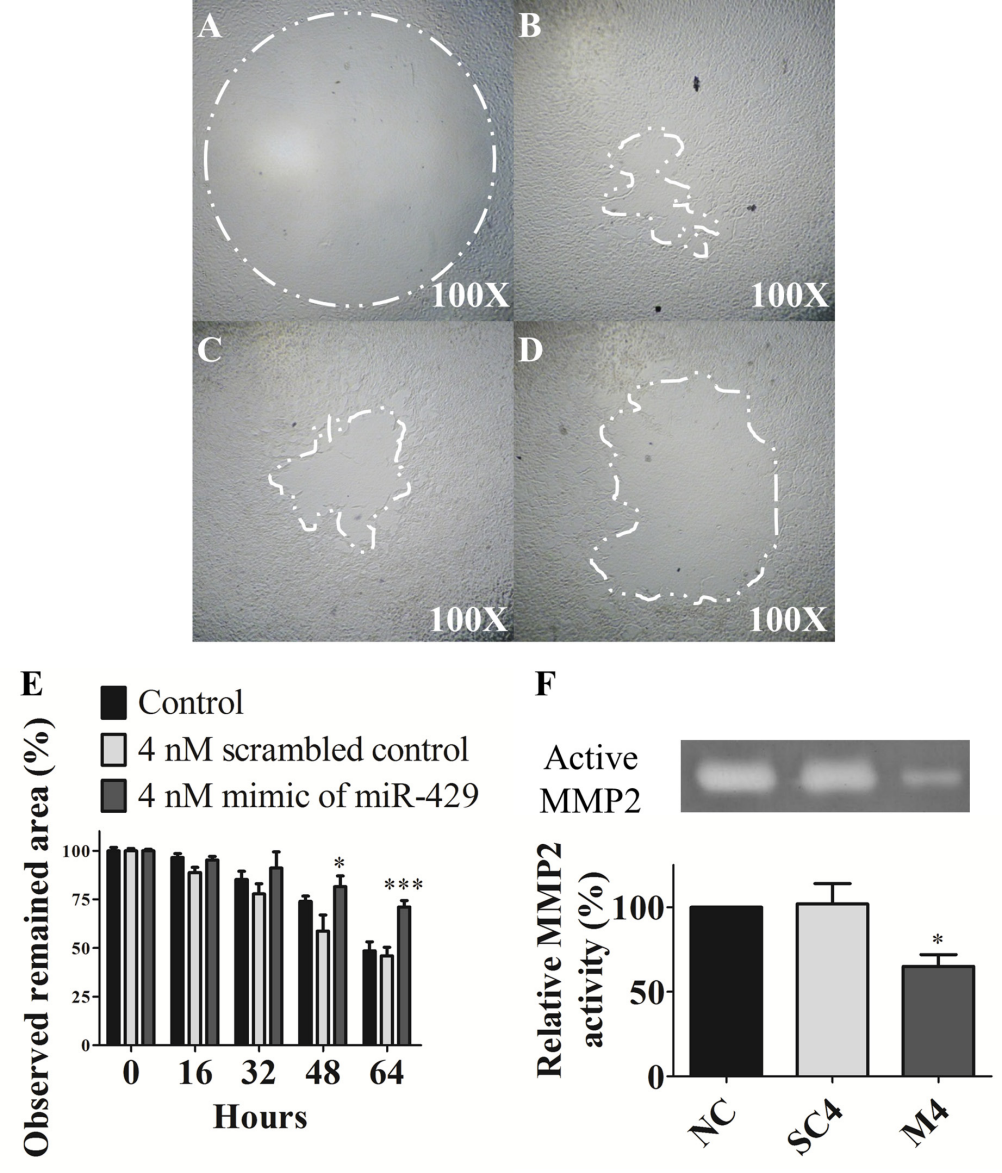
Invasive ability and MMP-2 activity are inhibited by exogenous miR-429 in T24 cells Invasion assay of (**A**) control 0 hour, (**B**) control 64 hours, (**C**) 4 nM scrambled control 64 hours, (**D**) 4 nM mimic of miR-429 64 hours in T24 cells and (**E**) statistical analysis was showed as histogram graph. (**F**) Zymography assay was performed in three groups, negative control (NC), 4 nM scrambled miRNA control (SC), and 4 nM mimic of miR-429 (M) from left to right lane in T24 cells and statistical analysis was showed as histogram graph (Student *t*-test, *p < 0.05; ***p < 0.001, Error bars = SD).

T24 cells were transfected and compared the invasive ability under matrix gel-coated wells. The observed remained area was remarkably larger in miR- 429 transfected T24 cells than non-treated controls at 48 hours (81.5% ± 5.5% vs 73.9% ± 2.8%, *p* < 0.05) and 64 hours (71.1% ± 3.3% vs 48.5% ± 4.7%, *p* < 0.001), individually. These results implicate that miR-429 has potential to inhibit cell invasive ability of UCC cells (Figure 4A–4E).

### MMP-2 activity is inhibited by exogenous miR-429 in T24 cells

The enzyme activity in 4 nM exogenous miR-429 transfected dramatically decreased the gelatin digestive ability than scrambled control (65.0% ± 7.0% vs 102.0% ± 12.0%, *p* < 0.05) and non-treated control groups in T24 cells (Figure 4F). These results suggest that miR-429 has potential to inhibit cell invasive ability through down-regulated the MMP-2 activity of UCC cells.

### E-cadherin knockdown reduces migratory ability of TSGH8301 cells

**Figure 5:**
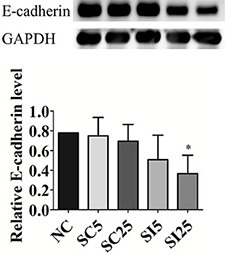
E-cadherin knockdown by siRNA in TSGH8301 cells Western blotting and histogram graph of E-cadherin. Histogram graph was performed in five groups, negative control (NC), 5 nM scrambled control (SC5), 25 nM scrambled control (SC25), 5 nM siRNA of E-cadherin (SI5), and 25 nM siRNA of E-cadherin (SI25) and GAPDH was used as loading control (Student *t*-test, *p < 0.05, Error bars = SD).

TSGH8301 cells were transfected with E-cadherin siRNA and scrambled control and assayed migratory ability by scratch healing assay. The E-cadherin expression marginal decreased with 5 nM (0.65 ± 0.32, *p* = 0.13) and significantly decreased with 25 nM (0.47 ± 0.24, *p* < 0.05) of E-cadherin siRNA when compared with control group (Figure 5).

**Figure 6:**
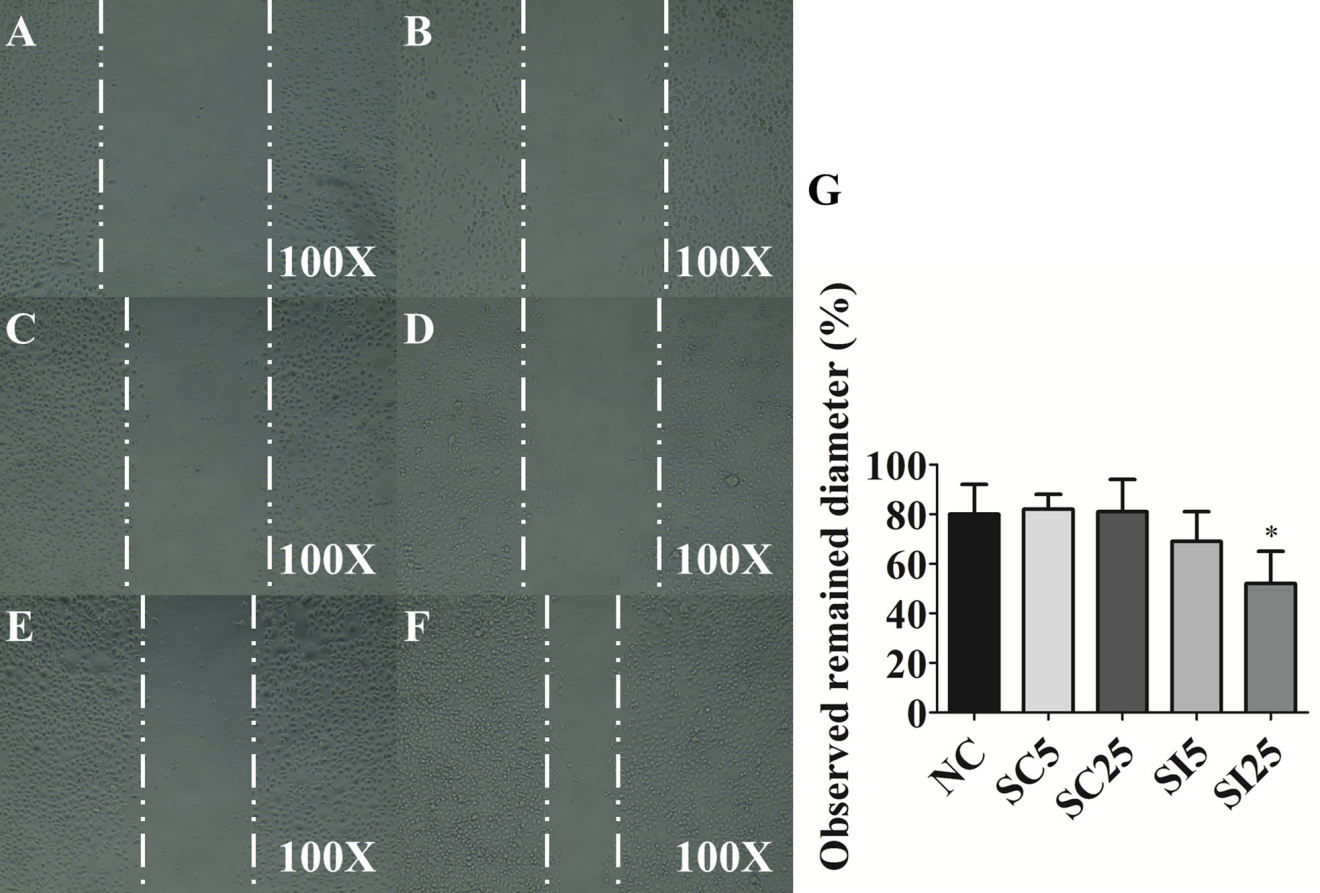
E-cadherin knockdown reduces migratory ability of TSGH8301 cells Scratch assay of observed remained diameter of (**A**) control 0 hour, (**B**) negative control (NC) 16 hours, (**C**) 5 nM scrambled control (SC5) 16 hours, (**D**) 25 nM scrambled control (SC25) 16 hours (**E**) 5 nM siRNA of E-cadherin (SI5) 16 hours (**F**) 25 nM siRNA of E-cadherin (SI25) 16 hours in TSGH8301 cells and (**G**) statistical analysis was showed as histogram graph (Student *t*-test, *p < 0.05, Error bars = SD).

The data showed that 5 nM E-cadherin siRNA (69.4% ± 11.6%, *p* = 0.26) had slightly inhibitory effect, and 25 nM E-cadherin siRNA (51.7% ± 13.3%, *p* < 0.05) had dramatically inhibitory effect on migration ability than 25 nM scrambled control (80.9% ± 12.6%, *p* = 0.90) when compared with non-treated control groups (79.8% ± 12.1%) (Figure 6). These results suggest that losses of E-cadherin enhanced the cell migratory ability in UCC cells.

## DISCUSSION

**Figure 7:**
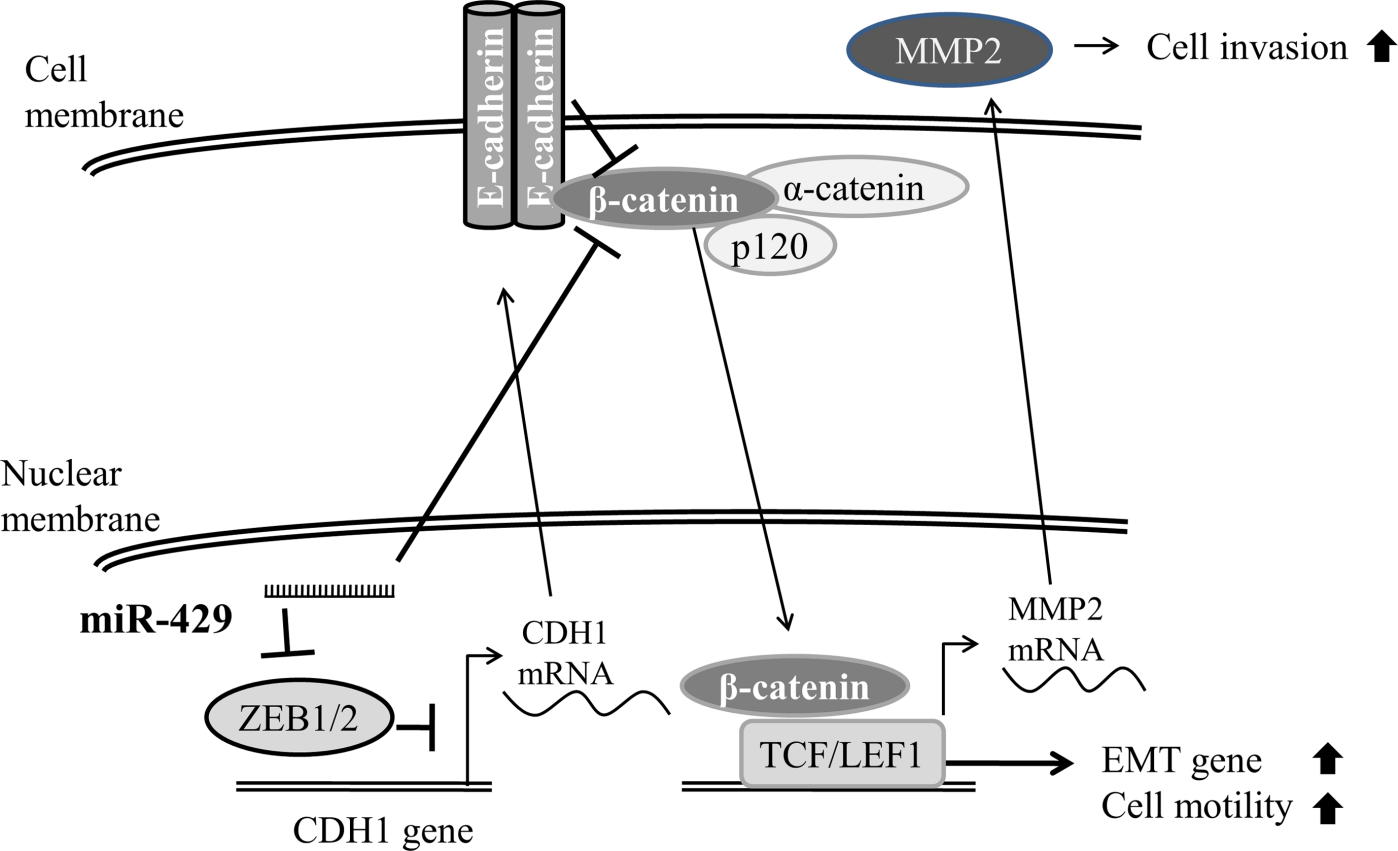
Schematic diagram of miR-429 in UCC cells Schematic diagram of miR-429 reduces ZEB1 and restores E-cadherin which results in downregulation of β-catenin and therefore inhibits cell migratory and invasive ability in bladder cancer cell.

To our knowledge, this is the first study addressed on the miR-429 role in bladder cancer. MiR-429, as other members of miR-200 family, act as a tumor suppressor roles during bladder cancer progression by reducing ZEB1, ZEB2 and restoring E-cadherin which results in downregulation of β-catenin and therefore inhibits cell migratory and invasive ability (Figure 7). Despite the clinicopathological role of miR-429 has not been surveyed in bladder cancer, downregulation of miR-429 has been found in several type of cancer including gastric cancer [12], breast cancer [13], renal cell carcinoma [14], colorectal carcinoma [15], and nasopharyngeal carcinoma [16]. However, contrary results are also reported for miR- 429 upregulated in some cancer types [17–19].

All members of miR-200 family have been considered to directly target ZEB1 and ZEB2, two strong transcriptional repressors which induce EMT by suppressing the expression of many epithelial genes, including E-cadherin [20]. And overexpression of miR- 200c has been proven to restore E-cadherin expression level [21]. Our results also indicated that miR-429 inhibits ZEB1 expression and therefore restores E-cadherin expression level in E-cadherin-negative T24 cells. Conversely, knockdown of E-cadherin also induces higher cell motility in intrinsic E-cadherin-positive TSGH8301. In sum, E-cadherin plays a pivotal role in the miR-429 mediated suppression of EMT signaling pathway [22]. In clinicopathological interpretation, increased ZEB1 and ZEB2 expression have been reported in human bladder carcinoma tissue [23], and ZEB2 represented as an independent factor of poor prognosis in the radiotherapy treated bladder cancer patients. Oppositely, losses of E-cadherin in bladder cancer specimens have been associated with the disease recurrence, metastasis and poor survival of bladder cancer patients [24–26].

Since the cytoplasmic domain of E-cadherin is anchored to the actin cytoskeleton by binding to α-catenin, β-catenin and p120-catenin and results in mechanical stability to the adherens junctions. Upon downregulation of E-cadherin, β-catenin is released from cell membrane and could be transferred into the nucleus to activate WNT target genes which leads to EMT of tumor cell and increases tumor invasiveness and/or metastasis. By degrading β-catenin that is released from E-cadherin, APC-axin-GSK-3β complex prevents β-catenin to be transferred into the nucleus and exert its transcriptional activity. Therefore, loss of E-cadherin increases the risk of nuclear translocation of β-catenin and thereby WNT pathway activity whilst the APC-axin-GSK-3β complex is impaired [27]. Accumulating β-catenin has been found to associate with increasing grade or stage of bladder cancer [28]. And β-catenin has been reported to transcriptionally induced MMP2 expression [29] which has been associated with induction of bladder cancer invasiveness [4]. Moreover, miR-200a has been reported to directly bind to 3′ UTR of *CTNNB1* (the gene that encodes β-catenin) which results in reduction of β-catenin and suppression of cell growth, migration and invasion [30]. Our results also indicate that miR-429 significantly reduces β-catenin protein expression and decreases MMP-2 activity on gelatin-zymography. In biological effects, miR-429 indeed reduced cell migratory and invasive abilities of bladder cancer cells. These results imply that miR-429 inhibits EMT phenotypes not only by targeting ZEB1, a potent transcriptional repressor of epithelial genes, but also by targeting β-catenin, a strong transcriptional activator of EMT genes.

Besides ZEB1, ZEB2, and β-catenin, miR-429 has been reported to target several other genes involved in several signaling pathways. For instance, miR-429 mediated suppression of EMT has also been correlated with miR-429 directly targets Onecut2 [31], BMI-1 and E2F3 [8], MiR-429 enhances cancer cell apoptosis under chemotherapy by targeting Bcl-2 [32] and AP-2α [33]. MiR-429 also can reduce cancer cell proliferation by targeting c-myc [34], TANK-binding kinase 1 [35] despite some controversial findings also reported [36]. In this study, we found that miR-429 can mediate suppression of EMT by reducing ZEB1 and β-catenin, but it is still not clear how miR-429 targeting network coordinated during bladder cancer progression. Further verifications of *in vitro* and *in vivo* molecular regulation should be performed in future studies. Preliminary study the expression of miR-429 in 76 clinical bladder cancer specimens in our laboratory revealed that low grade UCC had higher expression rate of miR-429 than high grade UCC and statistically related to recurrence-free survival and overall survival.

According to our results, miR-429 is significantly distinguishable between bladder cancer cells of different aggressiveness. It implicated that miR-429 is applicable for early detection of bladder cancer cell by using non-invasive *in situ* hybridization approach with miR-429 specific probe for recognizing miR-429-negative aberrant urothelial cells in urine sediments. On the other hand, comparison of miR-429 expression level using *in situ* hybridization with miR-429 specific probe should be helpful for prediction of disease progression of bladder cancer and miR-429 may be a potential target for therapeutic development of RNAi drug for bladder cancer.

Downregulation of other miR-200 family members, such as miR-200a, miR-200b and miR-200c, have been associated with poor overall or relapse-free survival of bladder cancer patients [37]. Therefore, miR-429 may also function as a useful prognostic marker during bladder cancer progression, and subsequent surveillance of the association between miR-429 expression profile in bladder cancer specimens and their clinical outcomes becomes necessary for evaluating clinical meaning of miR-429 in bladder cancer follow-up.

In summary, results of this study indicated that miR-429 was highly expressed predominately in low grade UCC cell lines. Furthermore, exogenous miR-429 decreased the cell migratory and invasive abilities through restoring the E-cadherin expression and inhibiting the MMP-2 activity in UCC cells. We propose that miR-429 might be used as a prognostic and predictive marker of invasiveness and metastasis in bladder cancer.

## MATERIALS AND METHODS

### Cell culture

Human UCC cell lines, T24 was purchased from ATCC (HTB-4^™^) and TSGH2010, TSGH8301, and TSGH9202 were established at our laboratory [38, 39]. All of them were cultured in Roswell Park Memorial Institute (RPMI)-1640 medium (Thermo Scientific, USA) supplemented with 10% fetal bovine serum (FBS) (Thermo Scientific, USA) and 100 U/mL penicillin, 50 μg/mL streptomycin (Sigma, USA) at 37°C in a 5% CO_2_ incubator.

### MiR expression in UCC cell lines detected by real-time quantitative polymerase chain reaction (Q-PCR)

Detection of miR expression level in various UCC cell lines was conducted by a three-step procedure using the ABI-9600 Instrument (ABI, USA). First, miR was extracted from cell lysate using NucleoSpin^®^ miR (MACHEREY-NAGEL, DE) according to the manufacturer's instructions. Second, cDNA was synthesized with Mir-X^™^ miR First-Strand Synthesis kit (Clontech Laboratories, USA) according to the manufacturer's instructions. Briefly, miR was polyadenylated with poly-A polymerase and reverse transcriptased with a specific miR primer and an mRQ 3′ primer. Third, 2 μL of the cDNA was amplified using 12.5 μL of the Smart Quant Green Master Mix with dUTP & ROX (PROTECH, UK) and 0.5 μL of 10 μM miR-specific primer and 0.5 μL of mRQ 3′ primer and 9.5 μL QH_2_O. The amplicon was detected by fluorescence using a specific miR-429 primer: forward primer 5′-TAATACTGTCTGGTAAAACCGT-3′ and an mRQ 3′ reverse primer. Other primers were shown in Supplementary Table 1. The fluorescence emitted after hybridization to the template DNA was measured by the ABI-9600 Instrument. In a separate real time PCR reaction, non-coding small nuclear RNA (snRNA) U6 was processed as a loading control. It served as a control for miR and relative quantification. All PCR reactions were performed with hot start activated with 15 min at 95°C, 40 cycles of 5 seconds at 95°C and 20 seconds at 60°C.

### MiR-429 transfection in T24 cells

Extrinsic miR-429 mimic and scrambled control (Phalanx Biotech, Taiwan) were transfected with jetPRIME^®^ (Polyplus-transfection USA) according to the manufacturer's instructions, individually. Briefly, 3 × 10^5^ T24 cells were seeded on the 6-well plates for 8 hours attachment. Culture medium was replaced to serum free medium for another 8 hours before transfection. MiR- 429 mimic and scrambled control were transfected for 18 hours and replaced to culture medium or serum free culture medium for another 18 or 48 hours. Transfected T24 cells were used in migration assay, invasion assay, and western blotting. In addition, serum free medium was used in zymography assay.

### E-cadherin knockdown in TSGH8301 cells

E-cadherin small interfering RNA (siRNA) and scrambled control (Dharmacon, USA) were transfected with jetPRIME^®^ (Polyplus-transfection USA) according to the manufacturer's instructions, individually. Briefly, 5 × 10^5^ TSGH8301 cells were seeded on the 6-well plates for 8 hours attachment. Culture medium was replaced to serum free medium for another 8 hours before transfection. E-cadherin siRNA and scrambled control were transfected for 18 hours and replaced to culture medium for another 18 hours. Transfected TSGH8301 cells were used in scratch assay and western blotting.

### Migration assay of UCC cell line T24

Cell migratory ability was measured by an Oris^™^ Pro Cell Migration Assay (Enzo Life Sciences, USA) according to the manufacturer's instructions. Briefly, the cell seeding stoppers were inserted in 96-well plate and 100 μL with 3 × 10^4^ T24 cells transfected with miR- 429 mimic or scrambled control, and non-treated control groups were seeded for 8 hours attachment, individually. The culture medium was refreshed and the stopper was removed as the experiment start time point 0 hour. The area of the seeding stopper was continuously recorded under light microscopy at 0, 8, 16, 24, 32, 40, and 48 hours. The area was measured from each picture using SPOT software (USA). Each experiment was repeated at least three times independently.

### Scratch healing assay of UCC cell line TSGH8301

Cell migratory ability was measured by scratch healing assay. Briefly, 5 × 10^5^ TSGH8301 cells transfected with E-cadherin siRNA or scrambled control, and non-treated control groups were seeded for 8 hours attachment, individually. The scratches were created by horizontal moved with 200 μL tips, washed with PBS twice, and refreshed the culture medium as the experiment start time point 0 hour. The diameter of scratch was recorded under light microscopy at 16 hours. The diameter was measured from each picture using SPOT software (USA). Each experiment was repeated at least three times independently.

### Western blotting of ZEB1, ZEB2, E-cadherin, β-catenin, and GAPDH expression

Cell mobility related and miR-429 targeting proteins, ZEB1, ZEB2, E-cadherin, and β-catenin were evaluated by western blotting, and glyceraldehyde 3-phosphate dehydrogenase (GAPDH) was used as loading control. Briefly, 1 × 10^6^ exponentially growing T24 and TSGH8301 cells of various groups were trypsinized and washed with PBS twice. Cells were resuspended in 100 mL of radioimmunoprecipitation assay (RIPA) buffer (PIERCE, USA) contained with cocktail protease inhibitor (Thermo Scientific, USA).

Protein (30 μg) was electrophoresed for 2 hour in 8% SDS-polyacrylamide gels and then transferred to polyvinylidene difluoride (PVDF) membranes (Millipore, USA) by electroblotter for 1 hour at 4°C with 100 voltage. Antibodies (Cell signaling, USA) raised against ZEB1, ZEB2, β-catenin, E-cadherin and GAPDH were diluted in TBST containing 5% BSA and membranes were incubated for 1 hour with gentle agitation. The blots were washed for three times with TBST and incubated with goat anti-rabbit antibody conjugated to horseradish peroxidase for 1 hour. After three successive washes with TBST, Western blotting chemiluminescence reagent (Thermo Scientific, USA) was used for protein detection.

### Invasion assay of T24 cells

Cell invasive ability was measured by an Oris^™^ Pro Cell Invasion Assay (Enzo Life Sciences, USA) according to the manufacturer's instructions. Briefly, 3 mg/mL of matrix gel (Trevigen, USA) in serum free culture medium was coated on the 96-well culture plate at 37°C for 30 minutes. After lower layer of matrix gel was coated, the cell seeding stoppers were inserted in 96-well plate and 100 μL medium with 3 × 10^4^ T24 transfected cells with miR-429 mimic or scrambled, and non-treated control groups were seeded for 8 hours attachment, individually. After stoppers were removed and washed the 96-well plate with PBS, 10 mg/mL of matrix gel in culture medium was coated above the attached cells at 37°C for 1 hour. After upper layer of matrix gel was coated, 100 μL of culture medium was added in 96-well plate as the experiment start time point 0 hour. The area of the seeding stopper was continuously recorded under light microscopy at 0, 16, 32, 48, and 64 hours. The area was measured from each picture using SPOT software (USA). Each experiment was repeated at least three times independently.

### Zymography assay of MMP-2 activity

MMP-2 activity was evaluated by gelatin zymography. Briefly, 40 mL of serum free culture medium was denatured in SDS buffer under non-reducing conditions (63 mM Tris HCl, 10% glycerol, 2% SDS, and 0.0025% bromophenol blue) without heating and electrophoresed for 3 hour in 10% SDS-polyacrylamide gels with 0.1% gelatin (Sigma, USA). The SDS-polyacrylamide gels were renatured by incubating the gel in renaturing buffer (2.5% Triton X-100) for 30 minutes twice and equilibrated in developing buffer (50 mM Tris base, 40 mM HCl, 200 mM NaCl, 5 mM CaCl_2_, and 0.02% Brij) 48 hours. The SDS-polyacrylamide gels were rinsed in staining buffer (0.1% Coomassie Brilliant Blue R-250, 40% ethanol, and 10% acetic acid) and followed in destaining buffer (10% ethanol and 7.5% acetic acid). Regions of protease activity appear as clear bands against a dark blue background.

### Statistical analysis

The expressions of miR and protein in cell lines were expressed as mean ± standard deviation. All the statistical analyses were performed using SPSS 16.0 and Excel 2007. All statistical tests and *p* values were two-sided and the level of significance was set at < 0.05 (*), < 0.01 (**), or < 0.001 (***).

## SUPPLEMENTARY MATERIALS TABLE AND FIGURE


