# Supplementary material for: MiR-429 reverses epithelial-mesenchymal transition by restoring E-cadherin expression in bladder cancer
Source: Oncotarget. 2016 Apr 2;7(18):26593–603. doi: 10.18632/oncotarget.8557 (PMC5042001; doi:10.18632/oncotarget.8557)
Supplement: Supplementary file 1 [file oncotarget-07-26593-s001.pdf]

MiR-429 reverses epithelial-mesenchymal transition by restoring E-cadherin expression in bladder cancer

Supplementary Materials

Supplementary Table S1: 42 EMT-related miRs' primer sequences

	primer sequence 5′ to 3′
miR-9	TCTTTGGTTATCTAGCTGTATGA
miR-17	CAAAGTGCTTACAGTGCAGGTAG
miR-20a	TAAAGTGCTTATAGTGCAGGTAG
miR-20b	CAAAGTGCTCATAGTGCAGGTAG
miR-21	TAGCTTATCAGACTGATGTTGA
miR-30a	TGTAAACATCCTCGACTGGAAG
miR-30b	TGTAAACATCCTACACTCAGCT
miR-30c	TGTAAACATCCTACACTCTCAGC
miR-30d	TGTAAACATCCCCGACTGGAAG
miR-30e	TGTAAACATCCTTGACTGGAAG
miR-31	AGGCAAGATGCTGGCATAGCT
miR-93	CAAAGTGCTGTTCGTGCAGGTAG
miR-101	TACAGTACTGTGATAACTGAA
miR-103	AGCAGCATTGTACAGGGCTATGA
miR-106a	AAAAGTGCTTACAGTGCAGGTAG
miR-106b	TAAAGTGCTGACAGTGCAGAT
miR-130a	CAGTGCAATGTTAAAAGGGCAT
miR-130b	CAGTGCAATGATGAAAGGGCAT
miR-145	GTCCAGTTTTCCCAGGAATCCCT
miR-181a	AACATTCAACGCTGTCGGTGAGT
miR-181b	AACATTCATTGCTGTCGGTGGGT
miR-181c	AACATTCAACCTGTCGGTGAGT
miR-181d	AACATTCATTGTTGTCGGTGGGT
miR-185	TGGAGAGAAAGGCAGTTCCTGA
miR-200b	TAATACTGCCTGGTAATGATGA
miR-200c	TAATACTGCCGGGTAATGATGGA
miR-203	GTGAAATGTTTAGGACCACTAG
miR-205	TCCTTCATTCCACCGGAGTCTG
miR-206	TGGAATGTAAGGAAGTGTGTGG
miR-221	AGCTACATTGTCTGCTGGGTTTC
miR-222	AGCTACATCTGGCTACTGGGT
miR-223	TGTCAGTTTGTCAAATACCCCA
miR-301a	CAGTGCAATAGTATTGTCAAAGC
miR-301b	CAGTGCAATGATATTGTCAAAGC
miR-302a	TAAGTGCTTCCATGTTTTGGTGA

miR-302b	TAAGTGCTTCCATGTTTTAGTAG
miR-338-3p	TCCAGCATCAGTGATTTTGTTG
miR-425	AATGACACGATCACTCCCGTTGA
miR-429	TAATACTGTCTGGTAAAACCGT
miR-489	GTGACATCACATATACGGCAGC
miR-519d	CAAAGTGCCTCCCTTTAGAGTG
miR-590-5p	GAGCTTATTCATAAAAGTGCAG

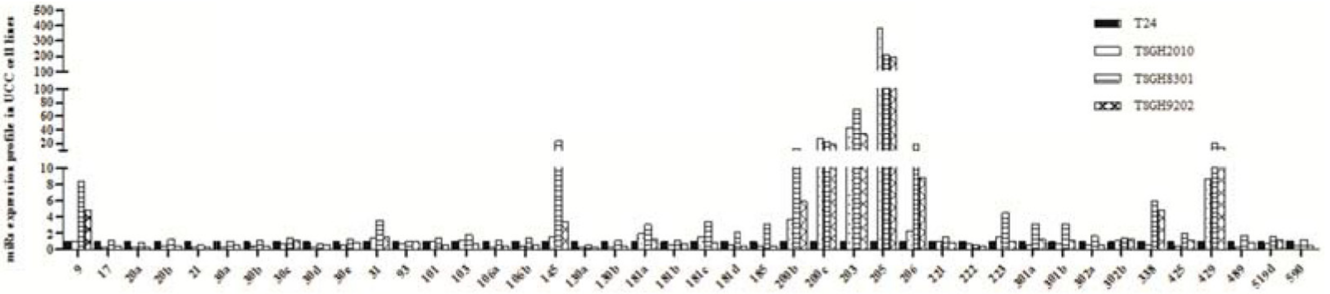

Supplementary Figure S1: 42 EMT-related miRs expression profiles in UCC cell lines.
